# Differences in dietary intake between users and non-users of online grocery shopping among Japanese adults: a cross-sectional study

**DOI:** 10.1186/s12937-025-01278-3

**Published:** 2025-12-30

**Authors:** Rei Fujiwara, Keiko Asakura, Haruhiko Imamura, Minami Sugimoto, Takehiro Michikawa, Yuji Nishiwaki

**Affiliations:** 1 Research Institute of Life Innovation, Toyo University, 1-7-11 Akabanedai, Kita-ku, Tokyo, 115-8650 Japan; 2 Department of Environmental and Occupational Health, School of Medicine, Toho University, 5-21-16 Omori-Nishi, Ota-ku, Tokyo, 143-8540 Japan; 3 Department of Preventive Medicine, School of Medicine, Toho University, 5-21-16 Omori-Nishi, Ota-ku, 143-8540 Tokyo, Japan; 4 Department of Community Well-being, School of Medicine, Toho University, 5-21-16 Omori-Nishi, Ota-ku, Tokyo, 143-8540 Japan; 5 Graduate School of Health and Nutrition Sciences, The University of Nagano, 8-49-7 Miwa, Nagano City, 380-8525 Japan

**Keywords:** Online grocery shopping, Food environment, Dietary intake, Diet quality

## Abstract

**Background:**

Although the use of Online Grocery Shopping (OGS) is growing rapidly, the effect of OGS use on dietary intake remains unclear. The purpose of this study was to examine the differences in the mean values of dietary intake (food group intake, nutrient intake, and diet quality) between OGS users and non-users. Furthermore, to investigate the potential influence of age, analyses were conducted separately for working-age adults (< 65 years) and older adults (≥ 65 years).

**Methods:**

In this cross-sectional study, 2,851 residents of Ota Ward, Tokyo were surveyed using a validated questionnaire to quantitatively assess the food group intake, nutrient intake, and diet quality score of OGS user and non-user groups. Analysis of covariance was conducted to compare adjusted means of food group intake, nutrient intake, and diet quality score between OGS users and non-users after adjusting for covariates. Analysis was performed by age in working-age adult (< 65y) and older (≥ 65 y) groups.

**Results:**

OGS users in the working-age adult group had higher intakes of potatoes, vegetables, fruits, protein, fiber, and several vitamins and minerals and a higher diet quality score than OGS non-users. In contrast, a relationship between OGS use and dietary intake was not apparent in the older group.

**Conclusions:**

OGS use was associated with a better dietary intake among the working-age adult group. Potential benefits of OGS use may not be fully realized among older populations, possibly due to differences in the rationale for OGS use and in digital literacy. Establishing a support system that enables older people to appropriately adopt and effectively utilize OGS may have the potential to serve as a tool that contributes to better food intake and diet quality among older adults.

**Supplementary Information:**

The online version contains supplementary material available at 10.1186/s12937-025-01278-3.

## Introduction

 Online Grocery Shopping (OGS) is a system in which customers use an online platform to purchase food and have it delivered to their home or designated location. Demand for and interest in OGS has increased dramatically, especially during and after the COVID-19 pandemic, and is expected to continue [1–4]. For example, the Food and Drug Administration (FDA) reported that online grocery sales in the United States increased by 55%, rising from $62 billion in 2019 to $96 billion in 2020 [5]. Similarly, the Ministry of Economy, Trade and Industry of Japan reported that the market size of online shopping for food (including beverages and alcoholic drinks) in Japan of 2.9299 trillion yen (this amount was approximately equivalent to 19.69 billion USD, based on the exchange rate of 148.77 yen per USD in September, 2023 [6]) in 2023 represented a 6.52% increase from the previous year [7].

This growing demand for OGS has raised interest in the use of OGS in nutritional research. Among findings, a US study reported that, compared to shopping at brick-and-mortar stores, use of OGS was associated with a higher proportion of consumer expenditure allocated to healthy food items, such as fruits and vegetables [8]; a lower proportion of consumer expenditure allocated to unhealthy food items, such as confectioneries [9, 10]; but may increase the purchase of unhealthy foods [11, 12]. Since these previous studies did not assess the dietary intake of OGS users, the question of whether OGS use affects dietary intake remains unclear. The close relationship between access to food and dietary intake is well known [13–16]. OGS has the potential to improve food access by providing alternative means of obtaining food, particularly for individuals with limited mobility, time constraints, or living in areas with poor access to physical stores. To our knowledge, however, little quantitative research has examined the relationship between OGS use and dietary intake.

Moreover, the relationship between OGS use and dietary behaviors may not be consistent across all population groups, particularly when differences in age are taken into account. In particular, the purpose of using OGS may vary by age group, and any such difference may contribute to age-related differences in the impact of OGS use on dietary intake. Although OGS users are generally considered to use the service for time savings and convenience [17, 18], these specific reasons appear inapplicable to older adults; rather, older adults tend to use OGS to alleviate the burden of grocery shopping as their physical function declines [19–21]. Understanding the effect of OGS on dietary intake therefore requires research which includes a focus on age.

The purpose of this study was to examine the differences in the dietary intake (food group intake, nutrient intake, and diet quality) between OGS users and non-users. Furthermore, to investigate the potential influence of age, analyses were conducted separately for working-age adults (< 65 years) and older adults (≥ 65 years).

## Methods

### Study design and participants

This cross-sectional study was based on data from a survey conducted by the local government of Ota Ward (one of the 23 wards of Tokyo) in October 2021. Ota Ward is located in the south-east of Tokyo, and had a population of approximately 700,000 at the time of the survey [22]. The primary objective of the survey was to collect data on lifestyles, dietary habits, and related factors to support the development of evidence-based local policies aimed at extending the healthy life expectancy of ward residents. Participants in the survey were randomly selected from a database of ward residents, consisting of 36,000 inhabitants aged in their 20s to 80s (2000 in each of the 18 districts of Ota Ward; approximately 5% of the population). They responded to a questionnaire regarding their health and lifestyle. In addition, 9,000 respondents (500 in each of the 18 districts) were given an additional dietary assessment using the Brief-type Self-administered Diet History Questionnaire (BDHQ) [23, 24]. Survey questionnaires were distributed to these selected residents via mail in September, 2021 and collected between October and December, 2021. A total of 2,851 participants answered the BDHQ (response rate: 31.7%), and after applying the exclusion criteria, 2,403 participants were included in the final analysis. In this study, we defined those aged 65 years or older as the older group and those working-age adults than 65 years as the working-age adult group. Although the definition of older adults varies depending on the context, we defined 65 years or older as older adults in accordance with the definition of older adults in the Annual Report on the Ageing Society published by the Cabinet Office, Government of Japan [25]. In addition, this cutoff is consistent with the OECD definition of the working-age population (15–64 years), thereby supporting the classification of individuals under 65 years as working-age adults.

The study was conducted with the support of the local government of Ota Ward and in accordance with the guidelines of the Declaration of Helsinki. The aim and protocol of the study were explained to the participants in a document attached to the questionnaire. In addition, the participants were informed that completion and return of the questionnaire was not mandatory, and that those who completed and returned it were considered to have consented to participation in the study. The research protocol was approved by the Ethical Review Committee of Toho University (approval of revised version: no. A_24024_A23035_A20057 on May 10, 2024; first approval: no. A20057 on September 17, 2020). The results of this study were reported in accordance with STROBE-nut [26].

### Definition of OGS use

The participants were asked to respond to the question, ‘Do you use online grocery services when purchasing food?’ in the lifestyle questionnaire. The response options in the questionnaire were ‘not using’, ‘using for less than half of all purchased foods’, ‘using for more than half of all purchased foods’ or ‘don’t know because I don’t purchase foods’. Two groups were created, an OGS non-user group (not using) and an OGS user group (using for less than half or more than half of all purchased foods). Participants who answered ‘don’t know because I don’t purchase foods’ were excluded from the analysis.

### Dietary assessment

Food group intake and nutrient intake were assessed using the BDHQ. This questionnaire has been described in detail elsewhere [23, 24]. Briefly, the BDHQ is a four-page fixed questionnaire developed based on the diet history method to evaluate the dietary habits of Japanese adults. This questionnaire asks about the frequency of intake of foods commonly consumed in Japan, general eating behaviors, and usual cooking methods, then estimates the intake of 58 foods and beverages in the previous month. Estimates of daily intakes of food, energy, and specific nutrients were calculated using an ad hoc computer algorithm for the BDHQ based on the 2015 version of the Standard Tables of Food Composition in Japan [27]. This ad hoc computer algorithm is a dedicated calculation program designed to convert reported intake frequencies into estimates of daily food, energy, and nutrient intakes using standardized portion-size assumptions and nutrient composition tables. A study evaluating the relative validity of BDHQ and 16-day weighed dietary record (DR) in Japanese participants aged 31–76 years found that validity was sufficient for food group intake (Spearman correlation coefficients: 0.44–0.48) [24] and for 42 nutrients (Pearson correlation coefficients: 0.54–0.56) [23] derived from BDHQ. Intakes of food groups and energy-non-producing nutrients were expressed as amounts per 1,000 kcal using the density method to allow comparison of dietary intake independently of individual differences in total energy intake [28]. Intakes of energy-producing nutrients – namely protein, total fat, saturated fatty acids, and carbohydrates - were expressed as a percentage of total energy intake, taking into account the amount of energy provided by each nutrient. Dietary supplements were not included in the dietary intake calculations because information on dietary supplements was not available.

### Assessment of diet quality

We used the Diet Quality Score for Japanese (DQSJ) as a measure of diet quality. Details of the DQSJ have been described elsewhere [29]. Briefly, the DQSJ consists of 10 items: 7 items that should be consumed in high amounts (1. fruits, 2. vegetables, 3. whole grains, 4. dairy products, 5. nuts, 6. legumes, 7. fish) and 3 that should be consumed in low amounts (8. red and processed meat such as beef and pork, 9. sugary drinks, 10. salt). The DQSJ score was calculated based on the distribution of intake stratified by sex. Each component was scored on a scale from 0 to 3 according to sex-specific quartiles of intake within the study population. For the seven components for which higher intake is considered favorable, 3 points were assigned to participants in the highest quartile, while for the three components for which lower intake is desirable, 3 points were assigned to those in the lowest quartile. Scores ranged from 0 to 30, with higher total scores indicating higher diet quality. The DQSJ has been shown to have moderate to strong correlations with major diet quality scores, including the Healthy Eating Index 2015, Healthy Eating Index 2010, Alternative Mediterranean Diet score and Dietary Approaches to Stop Hypertension (Spearman correlation coefficients: 0.52–0.84) [29]. In addition, evaluation of the relative validity of DQSJ scores estimated from the BDHQ against those derived from weighed DR demonstrated acceptable ranking ability (Pearson correlation coefficients: 0.59–0.61) [30]. In our study, DQSJ score was calculated based on the dietary intake estimated by the BDHQ.

### Other variables

In the lifestyle questionnaire, participants reported their age, sex, body height, body weight, education level (high school or lower/graduation from a two-year college or technical school, or university degree or higher), working status (working/non-working), working situation for those who were working (working hours per week, number of working days, working hours), cohabitants (living alone/living with cohabitants including children/living with cohabitants excluding children), current smoking status (smoking/not smoking), past medical history (none/present, including cancer, myocardial infarction or angina pectoris, difficulty walking, and stroke; selected because they may significantly impair mobility and independence, hindering daily activities such as grocery shopping), and perceived food environment (good/normal/poor). Further, frequency of housework and methods of obtaining health information were included as supplementary lifestyle variables. Perceived food environment was investigated using ‘Are the places where you usually purchase foods (e.g., stores or markets) easy to access from your home?’, with the response options ‘strongly agree,’ ‘agree,’ ‘not so much,’ ‘not at all,’ and ‘don’t know because I don’t purchase foods’. Respondents who answered ‘don’t know because I don’t purchase foods’ were excluded from the analysis. Perceived food environment was categorized into the three groups of ‘Good’ (if the participant answered ‘strongly agree’), ‘Normal’ (‘agree’), and ‘Poor’ (‘not so much’ or ‘not at all’). BMI was calculated as body weight (kg) divided by the square of body height (m).

### Statistical analysis

For participant characteristics, continuous variables were summarized as means and standard deviations (SDs), and categorical variables were presented as numbers and percentages. Differences between OGS user and non-user groups were compared using t-tests or χ² tests, as appropriate. Food group intake, nutrient intake, and diet quality score were summarized as adjusted means and standard errors (SE) using analysis of covariance. As potential confounders, we included age, sex, education level, working status, cohabitants, current smoking status, past medical history and food environment in the models. Analysis of covariance was conducted to compare adjusted means of food group intake, nutrient intake, and diet quality score between OGS users and non-users after adjusting for these covariates. The working-age adult and older age groups were analyzed separately to examine whether the associations differed by age group. All analyses were conducted using Stata/MP 16 for Windows (Stata Corp LLC, Texas, USA). Two-sided statistical tests were applied, and P values below 0.05 were considered statistically significant.

## Results

**Fig. 1:**
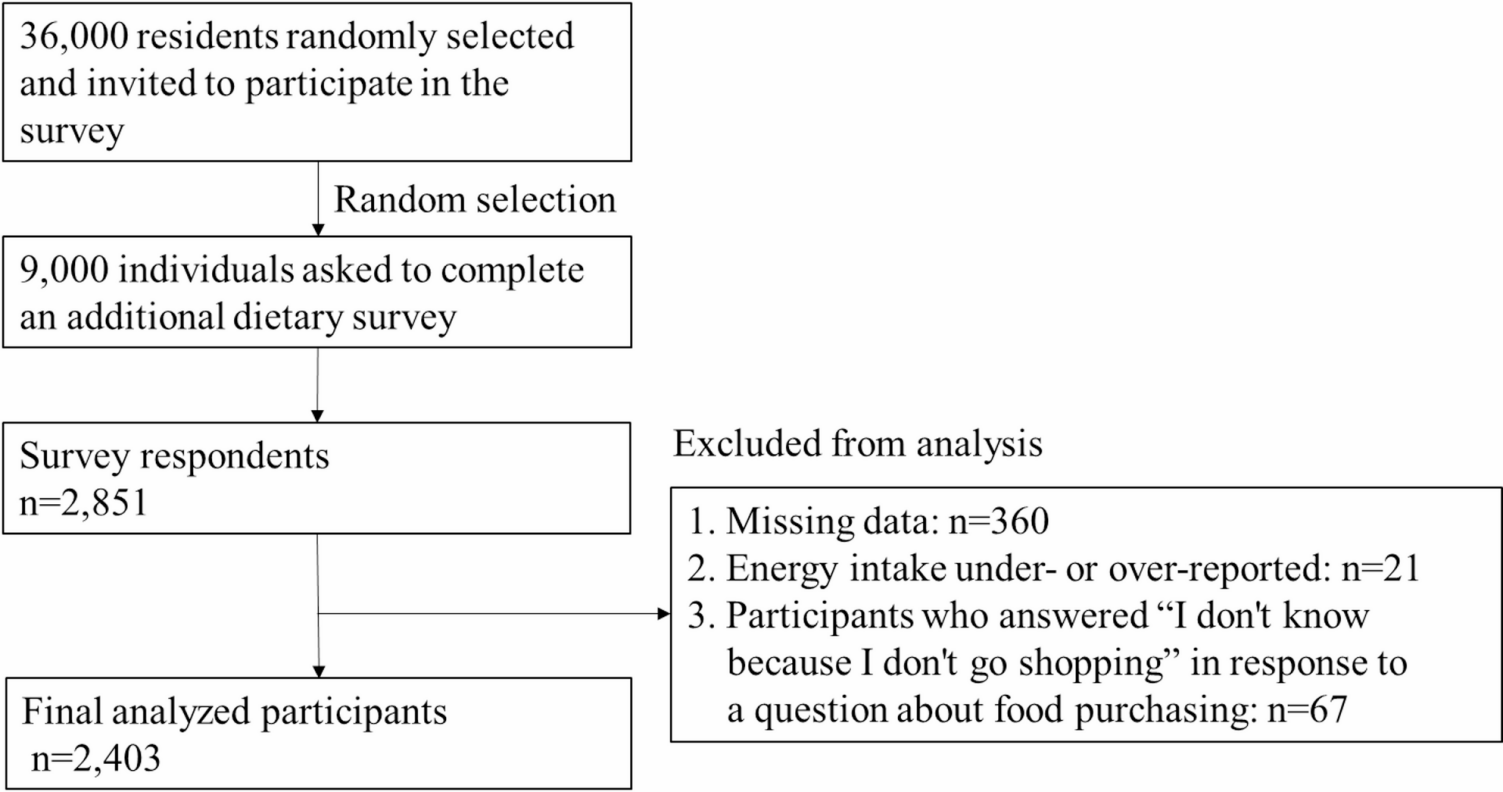
Flowchart of participant selection

Among the 2,851 respondents, those without necessary information (*n* = 360) and those suspected of underreporting (< 600 kcal) or overreporting (> 4500 kcal) their energy intake (*n* = 21) were excluded. In addition, participants who answered ‘don’t know because I don’t purchase foods’ to the question about OGS use and perceived food environment were also excluded (*n* = 67). As a result, the total number of participants included in the analysis was 2,403. The participant selection process is illustrated in Fig. 1.

The percentage of OGS users was 26.0% (*n* = 624) in all participants, and 31.8% (*n* = 422) in the working-age adult group and 18.8% (*n* = 202) in the older group.

Table 1 shows the basic characteristics of OGS users and non-users by age group. In both the working-age adult and older groups, OGS users had a significantly higher proportion of individuals with higher education, not working, and those living in a poor food environment compared to non-users. There were no significant differences in age, sex, body height, body weight, BMI, or energy intake. In the working-age adult group, OGS users had a significantly higher proportion of individuals living with cohabitants, including children, compared to non-users. In addition, working-age adult OGS users had significantly lower rates of smoking and past medical history than non-users. In the older group, OGS users had a significantly higher proportion of individuals with a past medical history compared to non-users.

**Table 1:** Characteristics of OGS users and non-users by age group (*n*=2,403)

Variable^a^	Mean, SD or *n* (%)													
	Working-age adult group							Older group						
	(<65 y/o)							(≥65 y/o)						
	OGS users			OGS non-users			*P* ^b^	OGS users			OGS non-users			*P* ^b^
	*n*= 422			*n*= 905				*n*=202			*n*=874			
Age, y/o	48.0	10.5		48.7	11.7		0.26	74.5	6.4		74.8		5.9	0.47
Sex							0.14							0.09
Male	155	(36.7)		371	(41.0)			78	(38.6)		395		(45.2)	
Female	267	(63.3)		534	(59.0)			124	(61.4)		479		(54.8)	
Height, cm (mean, SD)	163.4	8.0		163.4	8.2		0.94	158.6	9.6		158.9		8.9	0.64
Weight, kg (mean, SD)	60.0	12.5		60.1	12.1		0.90	57.5	11.8		58		10.9	0.59
BMI, kg/m^2^(mean, SD)	22.4	3.8		22.4	3.5		0.85	22.7	3.5		22.8		3.2	0.68
Education level							<0.001							0.003
High school or lower	85	(20.1)		270	(29.8)			86	(42.6)		475		(54.4)	
Graduated from a two-year college or technical school, or university degree or higher	337	(79.9)		635	(70.2)			116	(57.4)		399		(45.7)	
Working status							0.004							0.02
Working	318	(75.4)		743	(82.1)			46	(22.8)		275		(31.5)	
Non-working	104	(24.6)		162	(17.9)			156	(77.2)		599		(68.5)	
Working situation (those in employment only)	*n*= 318			*n*= 743				*n*=46			*n*=275			
Working hours per week, hours/week (mean, SD)^c^	38.6		16	38.8		14.5	0.81	24.9		16.8	26.1	14.5		0.60
Number of working days, days/week (mean, SD)	4.7		1.1	4.8		1	0.16	3.9		1.7	4.3	1.5		0.10
Working hours, hours/day (mean, SD)	8.0		2.4	7.9		2.4	0.82	6.1		2.3	6.0	2.5		0.82
Cohabitants							<0.001							0.95
Living alone	31		(7.4)	143		(15.8)		43		(21.3)	188	(21.5)		
Living with cohabitants excluding children	144		(34.1)	384		(42.4)		100		(49.5)	422	(48.3)		
Living with cohabitants including children	247		(58.5)	378		(41.8)		59		(29.2)	264	(30.2)		
Current smoking status							0.045							0.10
Smoking	52		(12.3)	150		(16.6)		9		(4.5)	68	(7.8)		
Not smoking	370		(87.7)	755		(83.4)		193		(95.5)	806	(92.2)		
Past medical history							0.04							0.03
Present	22		(5.2)	76		(8.4)		70		(34.7)	235	(26.9)		
None	400		(94.8)	829		(91.6)		132		(65.4)	639	(73.1)		
Perceived food environment							0.01							0.003
Poor	55		(13.0)	70		(7.7)		34		(16.8)	80	(9.2)		
Normal	203		(48.1)	480		(53.0)		112		(55.5)	490	(56.1)		
Good	164		(38.9)	355		(39.2)		56		(27.7)	304	(34.8)		
Intake energy (kcal/day)	1727		26	1722		18	0.88	1864		41	1871	20		0.88

*OGS* Online grocery shopping, *SD* Standard deviation, *y/o* years old, *BMI* Body mass index

^a^ Values are presented as either mean ± standard deviation or as numbers and percentages

^b^ Characteristics of OGS users and non-users were compared for the working-age adult and older groups separately using the t test for continuous variables and χ² test for categorical variables. *P* < 0.05 was considered to indicate statistical significance

^c^ Working hours per week =Number of working days (days/week) × Working hours (hours/day)

Table 2 shows the food group intake of OGS users and non-users by age group. In the working-age adult group, OGS users consumed significantly higher amounts of potatoes (23.8 ± 0.9 vs. 21.0 ± 0.6 g/1000 kcal/day, *p* = 0.02), total vegetables (141.9 ± 3.4 vs. 130.2 ± 2.3 g/1000 kcal/day, *p* = 0.01), and fruits (56.3 ± 2.4 vs. 48.8 ± 1.6 g/1000 kcal/day, *p* = 0.01) than non-users. Diet quality score was significantly higher for OGS users than non-users in the working-age adult group (12.5 ± 0.2 vs. 12.0 ± 0.1, *p* = 0.01). Among the older group, OGS users consumed significantly lower amounts of milk and dairy products (73.4 ± 4.1 vs. 85.5 ± 2.0 g/1000 kcal/day, *p* = 0.01) and 100% fruit juice (49.5 ± 2.7 vs. 55.6 ± 1.3 g/1000 kcal/day, *p* = 0.04) than non-users. There was no difference in diet quality score between users and non-users in the older group

**Table 2:** Food group intake and diet quality score of OGS users and non-users by age group (*n* = 2,403)

Variable^a^	Working-age adult group (< 65 y/o)					Older group (≥ 65 y/o)				
	OGS users		OGS non-users		*P* ^b^	OGS users		OGS non-users		*P* ^b^
	*n* = 422		*n* = 905			*n* = 202		*n* = 874		
	mean	SE	mean	SE		mean	SE	mean	SE	
Food group intake (g/1000 kcal/day)										
Rice	124.7	3.4	127.0	2.3	0.58	119.6	4.6	111.8	2.2	0.13
Bread	22.3	0.6	23.8	0.8	0.17	25.5	1.2	27.2	0.6	0.21
Noodles	41.1	1.5	44.1	1.0	0.11	41.8	2.1	40.5	1.0	0.59
Staple food ^c^	188.2	3.4	194.8	2.3	0.11	186.9	4.7	179.6	2.2	0.16
Potatoes	23.8	0.9	21.0	0.6	0.02	21.0	1.6	24.4	0.7	0.05
Pulses	38.3	1.4	36.0	0.9	0.19	42.1	2.0	41.9	0.9	0.90
Total vegetables	141.9	3.4	130.2	2.3	0.01	165.4	5.5	163.4	2.6	0.75
Fruits	56.3	2.4	48.8	1.6	0.01	77.1	4.3	78.3	2.0	0.80
Fish	38.8	1.1	36.3	0.7	0.06	49.8	2.0	50.9	0.9	0.64
Meat	47.9	1.1	47.1	0.8	0.56	41.7	1.4	41.6	0.7	0.96
Eggs	23.0	0.7	21.6	0.5	0.14	26.3	1.2	24.9	0.5	0.28
Milk and dairy products	72.3	2.9	66.1	2.0	0.08	73.4	4.1	85.5	2.0	0.01
Confectionaries	41.7	1.5	42.3	1.0	0.77	37.1	2.1	38.2	1.0	0.64
Sugar-sweetened beverages	29.2	3.2	32.6	2.1	0.39	21.8	3.6	24.6	1.7	0.48
Tea and coffee	56.8	4.4	50.4	3.0	0.24	47.6	5.7	41.0	2.7	0.30
100% Fruit juice	34.9	1.5	33.3	1.0	0.41	49.5	2.7	55.6	1.3	0.04
Diet quality score										
DQSJ	12.5	0.2	12.0	0.1	0.01	13.7	0.2	14.0	0.1	0.25

*OGS* Online grocery shopping, *DQSJ* Diet quality score for Japanese, *SE* standard error, *y/o* years old

^a^ Values are presented as adjusted means and standard errors

^b^ Characteristics of OGS users and non-users were compared for the working-age adult and older groups separately using analysis of covariance. Potential confounders included age, sex, education level, working status, cohabitants, current smoking status, past medical history and perceived food environment. *P* < 0.05 was considered to indicate statistical significance

^c^ Staple food (g/1000 kcal/day) = ((rice (g) + bread(g) + noodle (g))/1000 kcal)

Table 3 shows the nutrient intake of the OGS users and non-users by age group. In the working-age adult group, OGS users consumed significantly higher amounts of protein, total dietary fiber, thiamine, vitamin B2, niacin, vitamin B6, folate, and vitamin C, potassium, calcium, magnesium, iron, zinc, and copper compared to non-users. In the older group, the OGS users consumed significantly lower amount of calcium compared to the non-users.

**Table 3:** Nutrient intake of OGS users and non-users by age group (*n*=2,403)

Variable^a^	Working-age adult groupgroup (<65 y/o)					Older group (≥65 y/o)				
	OGS users		OGS non-users		*P* ^b^	OGS users		OGS non-users		*P* ^b^
	*n*= 422		*n*= 905			*n*=202		*n*=874		
	mean	SE	mean	SE		mean	SE	mean	SE	
Nutrient intake (unit)										
Protein (% energy/day)	15.6	0.1	15.1	0.1	0.01	16.6	0.2	16.9	0.1	0.28
Total fat (% energy/day)	29.4	0.3	28.7	0.2	0.05	29.1	0.4	29.3	0.2	0.72
Saturated fatty acid (% energy/day)	8.1	0.1	7.9	0.1	0.07	7.9	0.1	8.1	0.1	0.18
Carbohydrate (% energy/day)	49.0	0.4	49.4	0.3	0.42	49.2	0.6	49.2	0.3	1.00
Total dietary fiber (g/1000 kcal/day)	6.6	0.1	6.3	0.1	0.01	7.3	0.2	7.4	0.1	0.57
Vitamin A (µg RAE/1000 kcal/day)	410	11	391	7	0.17	438	21	453	10	0.52
Thiamine (mg/1000 kcal/day)	0.43	0.00	0.42	0.00	<0.001	0.46	0.01	0.46	0.00	0.54
Vitamin B2 (mg/1000 kcal/day)	0.75	0.01	0.72	0.01	0.01	0.81	0.01	0.84	0.01	0.09
Niacin (mg NE/1000 kcal/day)	9.9	0.1	9.6	0.1	0.01	10.1	0.2	10.3	0.1	0.30
Vitamin B6 (mg/1000 kcal/day)	0.71	0.01	0.68	0.01	0.004	0.78	0.01	0.76	0.01	0.23
Vitamin B12 (µg/1000 kcal/day)	5.0	0.1	4.7	0.1	0.07	6.1	0.2	6.4	0.1	0.21
Folate (µg/1000 kcal/day)	184	3	174	2	0.01	208	5	211	2	0.63
Vitamin C (mg/1000 kcal/day)	58	1	53	1	0.001	69	2	72	1	0.36
Vitamin D (mg/1000 kcal/day)	7	0	6	0	0.08	9	0	10	0	0.37
Sodium (mg/1000 kcal/day)	6	0.1	6	0	0.75	6.5	0.1	6.5	0.0	0.59
Potassium (mg/1000 kcal/day)	1421	18	1337	12	<0.001	1532	28	1570	13	0.24
Calcium (mg/1000 kcal/day)	304	5	284	3	0.001	340	8	356	4	0.04
Magnesium (mg/1000 kcal/day)	140	1	134	1	0.001	151	2	154	1	0.35
Iron (mg/1000 kcal/day)	4.4	0.1	4.2	0.0	0.001	4.9	0.1	4.9	0.0	0.95
Zinc (mg/1000 kcal/day)	4.5	0.0	4.4	0.0	0.02	4.6	0.1	4.7	0.0	0.77
Copper (mg/1000 kcal/day)	0.61	0.01	0.59	0.00	0.02	0.65	0.01	0.65	0.00	0.74

*OGS* online grocery shopping, *SE* standard error, *y/o* years old

^a^ Values are presented as adjusted means and standard errors

^b^ Comparisons of characteristics between OGS users and non-users were performed separately for the working-age adult and older groups using analysis of covariance. As potential confounders, we included age, sex, education level, working status, cohabitant, current smoking status, past medical history and perceived food environment. *P* < 0.05 was considered to indicate statistical significance

S1 Table shows frequency of housework in the OGS users and non-OGS users by age group. A higher proportion of OGS users reported that they ‘always’ engaged in housework compared to non-OGS users in both the working-age adult and older age groups. Specifically, 66.1% of working-age adult OGS users reported always doing housework, compared with 58.6% of non-users, and this pattern was even more pronounced in the older group (75.3% vs. 69.5%).

S2 Table shows the method used to obtain health information by age group. Among the older group, only 27.4% reported obtaining health information via the internet, versus 79.4% in the working-age adult group. Older adults relied more heavily on traditional media sources, such as television (73.5%) and newspapers (49.9%), whereas working-age adults were more likely to use digital sources.

S3 Table shows the differences in food group intake between working-age adults and older adults. Older adults generally consumed higher amounts of several food groups than working-age adults, including potatoes, fruits, fish, eggs, and 100% fruit juice, and they also had higher overall diet quality according to the DQSJ score.

S4 Table shows the differences in nutrient intake between the two age groups. Older adults had significantly higher intakes of multiple nutrients such as protein, total dietary fiber, several B vitamins, vitamin C, calcium, magnesium, iron, zinc, and copper compared with working-age adults.

S5-1 Table shows the relationship between food group intake (g/1000 kcal/day) and diet quality according to the DQSJ score. by perceived food environment. In the working-age adult group, those who reported a poor food environment had lower intakes of milk and dairy products and lower diet quality scores than those who perceived it as good. In the older group, participants who perceived their food environment as normal consumed more staple foods but fewer pulses and total vegetables than those who perceived it as good.

## Discussion

### Summary of key findings

The results of this study suggest a possible association between OGS use and healthier dietary intake among young adults. In contrast, among the older group, OGS use was not consistently associated with dietary intake, suggesting that the association between OGS use and dietary intake may differ by age group. To our knowledge, this is the first study to examine the association between OGS use and quantitatively assessed dietary intake by stratifying OGS users into working-age adult and older groups.

Previous studies of the relationship between OGS use and dietary habits reported that OGS use was beneficial in increasing the opportunity to purchase healthy foods such as vegetables, fruits and whole-grain products [8, 9]. In contrast, other studies noted that the convenience of being able to order food at any time may negatively impact dietary habits via the frequent ordering of foods with low nutritional value [10–12]. Our study showed that the OGS users in the working-age adult group had higher intakes of potatoes, vegetables and fruits than non-users as well as higher intakes of protein, fiber, several vitamins, and minerals. In addition, OGS users in working-age adult group also had significantly better diet quality scores. Moreover, we saw no significant difference in the intake of confectionery and sweetened beverages between the working-age adult OGS users and non-users. Our results therefore suggest that the use of OGS use among working-age adults may be associated with higher intakes of healthy foods such as vegetables and fruits, as well as with a more favorable overall diet quality. While the absolute differences in each nutrient or food group were modest, the consistent pattern across a range of dietary components suggests that these variations may represent meaningful differences in overall dietary intake at the group level. In contrast, no comparable differences were observed among older adults, underscoring a contrasting pattern between age groups and suggesting that the potential influence of OGS use on dietary intake may be more pronounced in working-age adults.

### Age-related differences in associations

The observed association between OGS use and healthy dietary intake among working-age adults may be attributable to the background characteristics of OGS users. In particular, our additional analysis of the frequency of housework among participants (S1 Table) showed that a higher proportion of OGS users than non-OGS users reported ‘always’ engaging in housework, suggesting that OGS users are more likely to engage in regular home cooking. Given that a higher proportion of OGS users have cohabiting family members, including children, and are more likely to be responsible for household tasks, it is possible that they pay greater attention to the healthfulness of their diet, which would then explain their own higher intake of vegetables and fruits and better diet quality score.

In contrast, different results were observed in the older group. We found no association between OGS use and dietary intakes except regarding the intake of milk and dairy products and of calcium. It has been reported that OGS use among older adults does not improve dietary diversity [31], and does not promote the purchase of healthier foods [32]. We propose two possible reasons for this: first, a significantly greater proportion of OGS users had a past medical history compared to non-OGS users. Older adults who use OGS may do so out of necessity rather than preference, owing to physical limitations that hinder their ability to shop for groceries. In contrast, working-age adult OGS users may use OGS to actively engage in healthy eating. These differences in the reason for using OGS may be related to age group-specific differences in the associations between OGS use and dietary intake. Second, the issue of digital literacy among older adults may have contributed to the inconsistent results. Compared to working-age adults, older adults are generally less familiar with the use of online services, which may have limited their ability to fully utilize OGS [33–35]. In our additional analyses on sources of health information (S2 Table), only 27.4% of older participants reported using the Internet to obtain health information, compared to 79.4% of the working-age adult group. These results indicate a disparity in digital literacy between working-age adults and older adults, which may in turn influence the extent to which OGS is utilized for a healthy diet.

### Characteristics and behaviors of OGS users

Characteristics of OGS users that were common to both the working-age adult and older groups included higher education level, being non-working, and living in a perceived poor food environment. The finding that OGS users were more educated than non-users is consistent with previous studies [36]. On the other hand, the association between working status and OGS was contrary to our assumptions: we had assumed that working individuals would be the main users of OGS, because consumers often use OGS for convenience and to save time. However, our results indicated a higher prevalence of non-workers among OGS users than non-OGS users. One possible explanation may lie in the structural characteristic of OGS, namely the necessity for users to be at home to receive deliveries, particularly fresh foods. In addition, previous research has indicated that the motivation for using OGS may extend beyond time savings to other purposes, such as purchasing rare foods not available in local markets [18]. Future research should therefore focus on the diversity of users’ lifestyles and motivations for OGS utilization, and undertake more detailed analyses of their sociodemographic characteristics and behavioral patterns. This information is essential for advancing the appropriate and effective use of OGS as a strategy to promote healthier food environments.

### Influence of perceived food environments

In our study, OGS users were significantly more likely to perceive their food environment as poor than non-users (Table 1). Additionally, participants who reported a poor food environment had lower intakes of foods such as pulses, and total vegetables (older group only), as well as a lower diet quality score (working-age adult group only), compared to those who perceived it as normal or good. These results suggest that OGS users who perceive their perceived food environment as poor may be using OGS to improve food access. The fact that the perceived food environment influences dietary habits is well known [37, 38]. For instance, individuals who perceive their food environment as favorable (i.e., feel that fruits and vegetables are easily accessible) tend to have higher actual intake of these foods [37]. Previous studies have suggested that OGS could serve as a stable source of healthy food supply [39–41]. In our study, OGS users also exhibited a tendency to utilize OGS as a means of overcoming disadvantages in their food environment. These findings suggest that such efforts may lead to improvements in dietary intake even when the surrounding food environment is not favorable.

In this study, older adults generally exhibited higher dietary intake and better diet quality according to the DQSJ score than working-age adults. This pattern is consistent with national data from the National Health and Nutrition Survey in Japan [42], showing that older adults consume more fruits and fish than younger adults. In addition, studies based on national datasets have similarly reported that older adults exhibit higher overall diet quality compared with younger populations [43]. However, these aggregate trends do not imply that all older adults maintain adequate nutritional status. Certain subgroups, such as those with mobility limitations or limited access to grocery stores, may still experience insufficient dietary intake or reduced dietary diversity. In our study, older adults who perceived their food environment as “poor” had lower intakes of legumes and vegetables than those who perceived their environment as “good” or “normal,” highlighting the presence of nutritional vulnerabilities within this age group. These findings underscore the need for future research to focus on such vulnerable subgroups and to examine whether OGS may serve as a supportive tool to help overcome food access constraints among older adults.

### Strengths and limitations

Our study has several strengths. First, the participants were randomly selected in an urban area based on the Basic Resident Register of Ota Word, Tokyo, where conducting surveys is generally considered challenging. Second, food group intake and nutrient intake were quantitatively assessed using the BDHQ and diet quality was evaluated using the DQSJ, both of which have been validated [23, 24, 29]. Additionally, this is the first study to identify a relationship between OGS use and quantitatively assessed food group intake and nutrient intake. Third, our study was based on a stratified analysis by age group, and demonstrated that the effects of OGS on dietary intake may differ between working-age adults and older adults.

Several limitations of this study should be mentioned. First, the participation rate was relatively low (2,851/9,000*100=31.7%). Additionally, since participation was voluntary, those who responded may have been more health-conscious, which could have introduced selection bias. A finding that both OGS users and non-users exhibit a high level of health consciousness might attenuate the observable differences in dietary intake between the two groups. Even under such circumstances, however, differences in dietary intake were observed between OGS users and non-users in the working-age adult group, suggesting that OGS use may be associated with dietary differences among working-age adults. In contrast, further investigation may be necessary to clarify the presence of differences among older adults. Second, as the study design was cross-sectional, our findings indicated associations rather than causality. Future interventional studies are needed to investigate the impact of introducing OGS on dietary behaviors. Third, the responses to questions regarding OGS use and food environment in this study were self-reported. Nevertheless, the question used to assess OGS use (“Do you use online grocery services when purchasing food?”) was simple and straightforward, making it unlikely that respondents misunderstood its intent. Furthermore, perceived food environment as measured in this study is believed to reflect aspects of food access that cannot be captured through objective indicators such as those derived using geographic information systems (GIS), supporting the validity of the measure used [44]. Finally, the lack of accounting for other means of food access may represent a potential unmeasured confounding factor. In recent years, the use of online food delivery services, namely the ordering of ready-to-eat meals from restaurants via digital platforms and other services has also increased worldwide. Food access is changing, particularly in high-income countries, including Japan [45]. Although this study provided meaningful insights, incorporating other means of food access into future analyses may allow for a more comprehensive understanding of the role and impact of OGS.

### Future research directions

Future studies are needed to build on the findings of this cross-sectional analysis. First, longitudinal or interventional research is required to clarify whether OGS use leads to subsequent improvements in dietary intake. Second, motivations and lifestyle characteristics associated with OGS use, particularly the differences observed between working-age and older adults should be examined in more detail. Third, digital literacy may play a critical role in determining whether older adults can effectively utilize OGS; therefore, research examining related barriers and potential support strategies is needed. Furthermore, the development of community-based digital support programs, as well as initiatives that encourage healthier food choices, would be important in enabling older adults to appropriately adopt and effectively use OGS. These efforts may, in turn, have the potential to contribute to better food intake and enhanced diet quality among older adults.

## Conclusions

Working-age adult OGS users had higher intakes of potatoes, vegetables and fruits than non-users, as well as higher intakes of protein, vitamins, and minerals. Their diet quality scores were also significantly higher than non-users. In contrast, these associations were not observed in the older group, suggesting that the relationship may vary by age. Although OGS has potential benefits, they may not be effectively utilized by older adults at present.

## Supplementary Information

Supplementary Material 1.

## Data Availability

The data are not publicly available due to privacy reasons.

## Abbreviations

OGS: Online grocery shopping
BDHQ: Brief-type, Self-administered Diet History Questionnaire
DR: Dietary record
DQSJ: Diet Quality Score for Japanese
GIS: Geographic information systems
