# Supplementary material for: Differences in dietary intake between users and non-users of online grocery shopping among Japanese adults: a cross-sectional study
Source: Nutr J. 2025 Dec 30;25:19. doi: 10.1186/s12937-025-01278-3 (PMC12866183; doi:10.1186/s12937-025-01278-3)
Supplement: Supplementary file 1 — Supplementary Material 1. [file 12937_2025_1278_MOESM1_ESM.docx]

Supplementary Materials

| S1 Table. Frequency of housework in OGS users and non-OGS users by age group (n=2,401). | | | | | | | | | | |
| --- | --- | --- | --- | --- | --- | --- | --- | --- | --- | --- |
| Variable^a^ | Working-age adult group  (<65 y/o) | | | | | Older group  (≥65 y/o) | | | | |
|  | OGS user | | OGS non- users | | *P*^b^ | OGS user | | OGS non- users | | *P*^b^ |
|  | n= 422 | | n= 905 | |  | n=202 | | n=872 | |  |
|  | n | % | n | % |  | n | % | n | % |  |
| Housework, n (%) |  |  |  |  | 0.044 |  |  |  |  | 0.032 |
| Always | 279 | 66.1 | 530 | 58.6 |  | 152 | 75.3 | 606 | 69.5 |  |
| Sometimes | 101 | 23.9 | 246 | 27.2 |  | 35 | 17.3 | 151 | 17.3 |  |
| Almost never | 30 | 7.1 | 96 | 10.6 |  | 15 | 7.4 | 84 | 9.6 |  |
| Not at all | 12 | 2.8 | 33 | 3.7 |  | 0 | 0 | 31 | 3.6 |  |
| OGS, online grocery shopping; y/o, years old ^a^ Variables expressed as number of people (%).  ^b^ Characteristics of OGS users and non-users were compared for the working-age adult and older groups separately using the t test for continuous variables and χ² test for categorical variables. *P* < 0.05 was considered to indicate statistical significance. | | | | | | | | | | |

| S2 Table. Method used to obtain health information by age group. | | | | | |
| --- | --- | --- | --- | --- | --- |
| Variable^a^ | Working-age adult group  (<65 y/o) | |  | Older group  (≥65 y/o) | |
|  | n=1,327 | |  | n=1,076 | |
|  | n | % |  | n | % |
| Books | 238 | 17.9 |  | 227 | 21.1 |
| Magazines & Weeklies | 181 | 13.6 |  | 202 | 18.8 |
| Newspapers | 239 | 18.0 |  | 537 | 49.9 |
| Television | 829 | 62.5 |  | 791 | 73.5 |
| Radio | 92 | 6.9 |  | 115 | 10.7 |
| Pamphlets and flyers | 76 | 5.7 |  | 113 | 10.5 |
| Internet/smart phone | 1,054 | 79.4 |  | 295 | 27.4 |
| Family | 299 | 22.5 |  | 210 | 19.5 |
| Friend, colleague | 277 | 20.9 |  | 208 | 19.3 |
| Hospital doctor or health care worker | 247 | 18.6 |  | 314 | 29.2 |
| Public health nurses, nutritionists, or dental hygienists in the ward | 24 | 1.8 |  | 19 | 1.8 |
| Health classes or events held in the ward | 5 | 0.4 |  | 5 | 0.5 |
| Not obtained in particular | 73 | 5.5 |  | 91 | 8.5 |
| y/o, years old ^a^ Variables expressed as number of people (%). Multiple answers allowed. | | | | | |

| Variable^a^ | Working-age adult group (<65 y/o) | | Older group  (≥65y/o) | | *P*^b^ |
| --- | --- | --- | --- | --- | --- |
|  | n= 1327 | | n= 1076 | |  |
|  | mean | SE | mean | SE |  |
| Food group intake (g/1000 kcal/day) |  |  |  |  |  |
| Rice | 118.0 | 2.6 | 123.5 | 3.1 | 0.27 |
| Bread | 25.1 | 0.7 | 24.7 | 0.8 | <0.001 |
| Noodles | 41.9 | 1.2 | 42.3 | 1.4 | <0.001 |
| Staple food ^c^ | 184.9 | 2.7 | 190.5 | 3.1 | 0.27 |
| Potatoes | 21.3 | 0.8 | 24.5 | 0.9 | 0.04 |
| Pulses | 37.9 | 1.1 | 40.5 | 1.3 | 0.22 |
| Total vegetables | 142.9 | 2.9 | 152.7 | 3.4 | 0.08 |
| Fruits | 58.9 | 2.1 | 68.5 | 2.5 | 0.02 |
| Fish | 40.5 | 1.0 | 46.5 | 1.1 | <0.001 |
| Meat | 44.6 | 0.8 | 45.1 | 1.0 | 0.76 |
| Eggs | 22.4 | 0.6 | 24.8 | 0.7 | 0.04 |
| Milk and dairy products | 74.0 | 2.3 | 75.9 | 2.7 | 0.67 |
| Confectionaries | 41.4 | 1.1 | 38.8 | 1.3 | 0.23 |
| Sugar-sweetened beverages | 28.0 | 2.3 | 28.4 | 2.7 | 0.94 |
| Tea and coffee | 49.5 | 3.4 | 45.8 | 4.0 | 0.57 |
| 100% Fruit juice | 40.7 | 1.3 | 46.0 | 1.6 | 0.03 |
| Diet quality score |  |  |  |  |  |
| DQSJ | 12.7 | 0.1 | 13.2 | 0.2 | 0.045 |
| DQSJ, Diet quality score for Japanese; SE, standard error; y/o, years old  ^a^ Values are presented as adjusted means and standard errors.  ^b^ Food group intakes were compared between working-age adult and older groups using analysis of covariance. Potential confounders included age, sex, education level, working status, cohabitants, current smoking status, past medical history and perceived food environment. *P* < 0.05 was considered to indicate statistical significance. ^c^ Staple food (g/1000 kcal/day) = ((rice (g)+ bread(g) + noodle (g))/1000 kcal) | | | | | |

S3 Table. Food group intake and diet quality score by the age groups (n=2,403)

| S4 Table. Nutrient intake by the age groups (n=2,403) | | | | | |
| --- | --- | --- | --- | --- | --- |
| Variable^a^ | Working-age adult group (<65 y/o) | | Older group  (≥65y/o) | | *P^b^* |
|  | n= 1327 | | n= 1076 | |  |
|  | mean | SE | mean | SE |  |
| Nutrient intake (unit) |  |  |  |  |  |
| Protein (% energy/day) | 15.6 | 0.1 | 16.5 | 0.1 | <0.001 |
| Total fat (% energy/day) | 28.8 | 0.2 | 29.5 | 0.3 | 0.12 |
| Saturated fatty acid (% energy/day) | 7.9 | 0.1 | 8.1 | 0.1 | 0.12 |
| Carbohydrate (% energy/day) | 48.9 | 0.3 | 49.7 | 0.4 | 0.24 |
| Total dietary fiber (g/1000 kcal/day) | 6.6 | 0.1 | 7.0 | 0.1 | 0.01 |
| Vitamin A (µg RAE/1000 kcal/day) | 415 | 10 | 428 | 12 | 0.51 |
| Thiamine (mg/1000 kcal/day) | 0.43 | 0.00 | 0.45 | 0.00 | 0.002 |
| Vitamin B2 (mg/1000 kcal/day) | 0.76 | 0.01 | 0.80 | 0.01 | 0.01 |
| Niacin (mg NE/1000 kcal/day) | 9.9 | 0.1 | 10.0 | 0.1 | 0.59 |
| Vitamin B6 (mg/1000 kcal/day) | 0.71 | 0.01 | 0.74 | 0.01 | 0.01 |
| Vitamin B12 (µg/1000 kcal/day) | 5.2 | 0.1 | 5.9 | 0.1 | <0.001 |
| Folate (µg/1000 kcal/day) | 188 | 3 | 197 | 3 | 0.08 |
| Vitamin C (mg/1000 kcal/day) | 59 | 1 | 65 | 1 | 0.002 |
| Vitamin D (mg/1000 kcal/day) | 8 | 0 | 8 | 0 | 0.68 |
| Sodium (mg/1000 kcal/day) | 6 | 0 | 6 | 0.1 | 0.01 |
| Potassium (mg/1000 kcal/day) | 1424 | 15 | 1485 | 17 | 0.03 |
| Calcium (mg/1000 kcal/day) | 310 | 4 | 329 | 5 | 0.01 |
| Magnesium (mg/1000 kcal/day) | 142 | 1 | 146 | 1 | 0.05 |
| Iron (mg/1000 kcal/day) | 4.4 | 0.0 | 4.6 | 0.1 | 0.01 |
| Zinc (mg/1000 kcal/day) | 4.4 | 0.0 | 4.6 | 0.0 | <0.001 |
| Copper (mg/1000 kcal/day) | 0.60 | 0.00 | 0.64 | 0.01 | <0.001 |
| SE, standard error; y/o, years old  ^a^ Values are presented as adjusted means and standard errors.  ^b^ Nutrient intakes were compared between working-age adult and older groups using analysis of covariance. Potential confounders included age, sex, education level, working status, cohabitants, current smoking status, past medical history and perceived food environment. *P* < 0.05 was considered to indicate statistical significance. | | | | | |

| S5-1 Table. Relationship between dietary intake by perceived food environment among the working-age adult group (n=1,327). | | | | | | | |
| --- | --- | --- | --- | --- | --- | --- | --- |
|  | Food environment | | | | | | |
| Variable^a^ | Poor n＝125 | | Normal n＝683 | | Good n＝519 | | *P^b^* |
|  | means | SE | means | SE | means | SE |  |
| Food group intake (g/1000 kcal/day) | |  |  |  |  |  |  |
| Rice | 131.7 | 6.0 | 125.8 | 2.6 | 125.5 | 3.0 | 0.48 |
| Bread | 23.8 | 1.5 | 23.2 | 0.7 | 23.3 | 0.8 | 0.85 |
| Noodle | 45.9 | 2.7 | 43.5 | 1.2 | 41.9 | 1.3 | 0.15 |
| Staple foods^c^ | 201.3 | 6.2 | 192.7 | 2.7 | 190.6 | 3.1 | 0.18 |
| Potatoes | 19.3 | 1.7 | 22.4 | 0.7 | 21.9 | 0.8 | 0.46 |
| Pulses | 35.8 | 2.5 | 36.6 | 1.1 | 37.2 | 1.2 | 0.55 |
| Total vegetables | 127.7 | 6.2 | 135.1 | 2.7 | 134.0 | 3.1 | 0.53 |
| Fruits | 43.3 | 4.4 | 51.4 | 1.9 | 52.8 | 2.1 | 0.09 |
| Fish | 33.4 | 1.9 | 37.6 | 0.8 | 37.3 | 0.9 | 0.23 |
| Meat | 48.1 | 2.0 | 47.9 | 0.9 | 46.5 | 1.0 | 0.30 |
| Eggs | 22.2 | 1.3 | 21.4 | 0.6 | 22.8 | 0.7 | 0.29 |
| Milk and dairy products | 55.5^a^ | 5.3 | 67.3^a,b^ | 2.3 | 72.1^b^ | 2.6 | 0.01 |
| Confectionaries | 44.4 | 2.7 | 41.0 | 1.1 | 43.0 | 1.3 | 0.80 |
| Sugar-sweetened beverages | 30.1 | 5.7 | 31.0 | 2.5 | 32.5 | 2.8 | 0.70 |
| Tea and coffee | 56.5 | 8.0 | 51.3 | 3.5 | 52.9 | 4.0 | 0.91 |
| 100% Fruit juice | 29.3 | 2.7 | 32.4 | 1.2 | 36.8 | 1.3 | 0.79 |
| Diet quality score |  |  |  |  |  |  |  |
| DQSJ | 11.5^a^ | 0.3 | 12.0^a,b^ | 0.1 | 12.5^b^ | 0.2 | 0.002 |
| DQSJ, Diet quality score for Japanese; SE, standard error ^a^ Values are presented as adjusted means and standard errors. ^b^ *P* value by analysis of covariance was analyzed. Potential confounders included age, sex, education level, working status, cohabitants, current smoking status and past medical history. *P* < 0.05 was considered to indicate statistical significance. Different letters between food environment groups indicate statistically significant differences, as determined by the Tukey-Kramer test. ^c^ Staple foods (g/1000 kcal/day) = ((rice (g)+ bread(g) + noodle (g))/1000 kcal) | | | | | | | |

| S5-2 Table. Relationship between dietary intake by perceived food environment among the older age group (n=1,076). | | | | | | | |
| --- | --- | --- | --- | --- | --- | --- | --- |
|  | Food environment | | | | | | |
| Variable^a^ | Poor  n＝114 | | Normal  n＝602 | | Good n＝360 | | *P^b^* |
|  | means | SE | means | SE | means | SE |  |
| Food group intake (g/1000 kcal/day) | |  |  |  |  |  |  |
| Rice | 113.1 | 6.1 | 116.2 | 2.7 | 108.4 | 3.4 | 0.19 |
| Bread | 28.0 | 1.6 | 26.8 | 0.7 | 26.7 | 0.9 | 0.59 |
| Noodle | 43.5 | 2.8 | 41.5 | 1.2 | 38.7 | 1.6 | 0.10 |
| Staple foods^c^ | 185.2^a,b^ | 6.2 | 184.7^a^ | 2.7 | 173.5^b^ | 3.5 | 0.03 |
| Potatoes | 23.4 | 2.1 | 23.7 | 0.9 | 24.0 | 1.2 | 0.84 |
| Pulses | 37.7^a,b^ | 2.6 | 40.5^a^ | 1.1 | 45.6^b^ | 1.5 | 0.002 |
| Total vegetables | 162.5^a,b^ | 7.3 | 158.9^a^ | 3.2 | 172.3^b^ | 4.1 | 0.045 |
| Fruits | 77.9 | 5.7 | 76.8 | 2.5 | 80.2 | 3.2 | 0.57 |
| Fish | 53.1 | 2.6 | 50.5 | 1.1 | 50.2 | 1.5 | 0.47 |
| Meat | 41.0 | 1.8 | 41.5 | 0.8 | 41.9 | 1.0 | 0.65 |
| Eggs | 24.5 | 1.5 | 25.3 | 0.7 | 25.0 | 0.9 | 0.90 |
| Milk and dairy products | 85.9 | 5.5 | 82.5 | 2.4 | 83.6 | 3.1 | 0.90 |
| Confectionaries | 34.5 | 2.7 | 37.3 | 1.2 | 40.2 | 1.5 | 0.06 |
| Sugar-sweetened beverages | 21.2 | 4.7 | 22.8 | 2.0 | 27.0 | 2.6 | 0.16 |
| Tea and coffee | 45.0 | 7.6 | 40.9 | 3.3 | 43.5 | 4.3 | 0.97 |
| 100% Fruit juice | 56.8 | 3.5 | 53.5 | 1.5 | 55.3 | 2.0 | 0.51 |
| Diet quality score |  |  |  |  |  |  |  |
| DQSJ | 13.7 | 0.3 | 13.9 | 0.1 | 14.1 | 0.2 | 0.29 |
| DQSJ, Diet quality score for Japanese; SE, standard error ^a^ Values are presented as adjusted means and standard errors. ^b^ P value by analysis of covariance was analyzed. Potential confounders included age, sex, education level, working status, cohabitants, current smoking status and past medical history. P < 0.05 was considered to indicate statistical significance. Different letters between food environment groups indicate statistically significant differences, as determined by the Tukey-Kramer test. ^c^ Staple foods (g/1000 kcal/day) = ((rice (g)+ bread(g) + noodle (g))/1000kcal) | | | | | | | |
